# *mcr-1−*Harboring *Salmonella enterica* Serovar Typhimurium Sequence Type 34 in Pigs, China

**DOI:** 10.3201/eid2302.161543

**Published:** 2017-02

**Authors:** Linxian Yi, Jing Wang, Yanling Gao, Yiyun Liu, Yohei Doi, Renjie Wu, Zhenling Zeng, Zisen Liang, Jian-Hua Liu

**Affiliations:** South China Agricultural University, Guangzhou, China (L. Yi, J. Wang, Y. Liu, R. Wu, Z. Zeng, Z. Liang, J.-H. Liu);; Henan General Institute of Animal Drugs and Feedstuff, Zhengzhou, China (Y. Gao);; University of Pittsburgh Medical Center, Pittsburgh, Pennsylvania, USA (Y. Doi)

**Keywords:** mcr-1 gene, colistin, Salmonella enterica serovar Typhimurium ST34, bacteria, antimicrobial resistance, clonal spread, sequence type, pigs, slaughter, food safety, zoonoses, China

## Abstract

We detected the *mcr-1* gene in 21 (14.8%) *Salmonella* isolates from pigs at slaughter; 19 were serovar Typhimurium sequence type 34. The gene was located on IncHI2-like plasmids that also harbored IncF replicons and lacked a conjugative transfer region. These findings highlight the need to prevent further spread of colistin resistance in animals and humans.

Since our report of emergence of a plasmid-mediated colistin resistance mechanism involving the *mcr-1* gene in *Escherichia coli* (*1*), *mcr-1* has been found in >30 countries in 5 continents in <1 year (*2*). *E. coli* is the main host of *mcr-1*, although several other *Enterobacteriaceae*, including *Salmonella*, have also been implicated as carriers of this gene (*3**,**4*). We screened pigs at slaughter for *Salmonella* isolates to elucidate the epidemiology of *mcr-1* in this major foodborne pathogen, which a serious public health problem.

## The Study

During July 2013–May 2014, a total of 1,780 cecum samples were obtained from pigs at 5 slaughter houses in southern and central China. Samples were incubated in buffered peptone water for 20 h and then inoculated into chromogenic medium selective for *Salmonella* spp. (CHROMagar Microbiology, Paris, France) for 24 h. Suspected *Salmonella* colonies were selected (1 isolate was selected from each sample) and identified by using PCR for detection of the *invA* gene.

We obtained 142 *Salmonella* isolates and screened them for *mcr-1* by using PCR and sequencing with primers described (*1*); 21 (14.8%) were positive for *mcr-1*. These isolates were serotyped as described (*5*). We determined the clonal relationship of *mcr-1*–positive isolates by using pulsed-field gel electrophoresis (PFGE) with *Xbal*I restriction enzyme and identified sequence types (STs) of these isolates by using multilocus sequence typing (http://mlst.warwick.ac.uk/mlst/dbs/Senterica). 

Most of the isolates had indistinguishable PFGE patterns and were clonally related to serovar Typhimurium ST34 (n = 19), which was the dominant type (Table). ST34 is common in isolates from humans in China and other countries and has been linked to food-producing animals (*6**,**7*).

**Table:** Characteristics of *mcr-1*–positive *Salmonella* isolates from pigs at slaughter, China, 2013–2014*

Strain	Serovar	PFGE type	Sequence type	S1-PFGE, plasmid, kb	Colistin MIC, mg/L	Polymyxin B MIC, mg/L	Other antimicrobial drug resistance†	Resistance gene‡
SH149	Typhimurium	A5	34	≈180	2	4	AMP, STR, GEN, FFC, SXT, TET	*floR*, *oqxAB*
SH143	Typhimurium	A1	34	≈180	2	4	AMP, STR, GEN, FFC, SXT, TET	*floR*, *oqxAB*
SH138	Typhimurium	A1	34	≈180	2	4	AMP, STR, GEN, FFC, SXT, TET	*floR*, *oqxAB*
SH93	Typhimurium	A1	34	≈180	2	8	FFC, SXT, TET	*floR*, *oqxAB*
SH33	Typhimurium	A1	34	≈110	2	4	AMP, STR, SXT, TET	*oqxAB*
SH137	Typhimurium	A1	34	≈180	2	8	AMP, STR, GEN, FFC, SXT, TET	*floR*, *oqxAB*
SA316	Typhimurium	A1	34	≈180	2	8	AMP, STR, GEN, FFC, SXT, TET	*floR*
SH127	Typhimurium	A1	34	≈180	2	4	AMP, STR, GEN, FFC, SXT, TET	*floR*, *oqxAB*
SH128	Typhimurium	A1	34	≈180	2	4	AMP, STR, GEN, FFC, SXT, TET	*floR*, *oqxAB*
SH133	Typhimurium	A1	34	≈180	2	4	AMP, STR, GEN, FFC, SXT, TET	*floR*, *oqxAB*
SH134	Typhimurium	A1	34	≈180	2	4	AMP, STR, GEN, FFC, SXT, TET	*floR*, *oqxAB*
SH17	Typhimurium	A1	34	≈180	2	4	AMP, STR, GEN, FFC, SXT, TET	*floR*, *oqxAB*
SH239	Typhimurium	A1	34	≈180, ≈138	2	8	AMP, STR, GEN, FFC, SXT, TET	*floR*
SH271	Typhimurium	A1	34	≈180	2	8	AMP, STR, GEN, FFC, SXT, TET	*floR*, *oqxAB*
SH83	Typhimurium	A1	34	≈180	2	4	AMP, STR, GEN, FFC, SXT, TET	*floR*, *oqxAB*
SH205	Typhimurium	A1	34	≈180	2	8	AMP, STR, GEN, FFC, SXT, TET	*floR*, *oqxAB*
SH39	Typhimurium	A2	34	≈180	2	4	AMP, STR, GEN, FFC, SXT, TET	*floR*, *oqxAB*
SH178	Typhimurium	A1	34	≈180	2	4	AMP, STR, GEN, FFC, SXT, TET	*floR*, *oqxAB*
SH175§	Typhimurium	A4	34	≈180	2	8	AMP, STR, GEN, **FFC,** SXT, TET	***floR*, *oqxAB***
SH36§	Heidelberg	B	ND	≈180	2	8	STR, **FFC**, SXT	***floR*, *oqxAB***
Z4P319S§	London	C	ND	≈180	1	2	AMP, STR, GEN, **FFC**,CIP, SXT, TET	***floR*, *oqxAB***

*According to recommendations of the joint Clinical and Laboratory Standards Institute (CLSI)–European Committee on Antimicrobial Susceptibility Testing (EUCAST) and the Polymyxin Breakpoints Working Group (http://www.eucast.org/), the International Organization for Standardization standard broth microdilution method (20776–1) was used to determine colistin MICs for these strains. Antimicrobial susceptibility was determined and evaluated according to CLSI document no. M100-S25 (http://shop.clsi.org/site/Sample_pdf/M100S25_sample.pdf). Resistance to florfenicol was interpreted according to EUCAST clinical breakpoints and epidemiological cutoff values (>16 mg/L) (http://mic.eucast.org/Eucast2/). MICs of olaquindox for all isolates, except SH36, were >64 mg/L. AMP, ampicillin; CIP, ciprofloxacin; FFC, florfenicol; GEN, gentamicin; ND, not determined; STR, streptomycin; SXT, sulfamethoxazole/trimethoprim; TET, tetracycline. †Boldface indicates resistance phenotypes transferred to a recipient by transformation. ‡Boldface indicates resistance genes transferred to a recipient by transformation. §Original strains of transformants.

We determined MICs for 12 antimicrobial drugs for all *mcr-1–*positive isolates by using agar dilution methods or a broth microdilution method. Colistin showed MICs of 1–2 mg/L for these isolates (Table). More than 80% of the isolates were also resistant to ampicillin, streptomycin, florfenicol, tetracycline, sulfamethoxazole/trimethoprim, and gentamicin; 76.2% showed reduced susceptibility (MIC >0.06 mg/L) to ciprofloxacin.

We identified 2 lipopolysaccharide regulatory genes (*pmrA* and *pmrB*) by using and PCR and sequencing (*8*). Plasmid-mediated resistance genes *floR* (florfenicol resistance) and *oqxAB* (olaquindox and ciprofloxacin resistance) were detected by using PCR. None of the 21 isolates had mutations in the *pmrA* and *pmrB* genes, which are associated with colistin resistance in *Salmonella* isolates. A total of 20 and 19 isolates had the *floR* and *oqxAB* genes, respectively. *Salmonella* Typhimurium ST34 has also been associated with the spread of the *oqxAB* gene in human clinical isolates in China (*9*), and *Salmonella* Typhimurium ST34 coproducing *oqxAB* or *floR* genes has also been isolated from humans in Europe and Canada (*4**,**10*).

To determine the genetic context of *mcr-1*, we used PCR mapping with primers ISAP-F (5′-CGAAGCACCAAGACATCA-3′) and MCR-R (5′-CCACAAGAACAAA CGGACT-3′). Insertion sequence IS*Apl1* was found upstream of *mcr-1* for all isolates.

We determined transferability and location of *mcr-1* by using conjugation, transformation, and hybridization (with S1-PFGE nuclease). These procedures showed that *mcr-1* genes were located on an ≈180-kb plasmid, except for those in strains SH33 (≈110-kb plasmid) and SH239 (≈180-kb plasmid and ≈138-kb plasmid) (Table). However, the *mcr-1* gene could not be transferred to *E. coli* C600 by conjugation.

We then randomly selected 6 *mcr-1*–positive *Salmonella* isolates (SH138, SH149, SH175, SH36, SH39, and Z4P319S) for chemical transformation, which was successful for SH175, SH36, and Z4P319S. S1-PFGE confirmed that transformants harbored single plasmids and had 8–16-fold higher MICs for colistin than the recipient *E. coli* DH5α. The *floR* and *oqxAB* genes were also transferred with the *mcr-1* gene.

We used Hiseq Technology (Illumina, San Diego, CA, USA) to sequence plasmid DNA purified from transformants of *Salmonella* Heidelberg SH36 and *Salmonella* London Z4P319S and genomic DNA extracted from the original isolate (*Salmonella* Typhimurium SH138, which was selected as a representative ST34 strain). We assembled sequence reads were assembled into contigs by using SOAPdenovo version 2.04 (http://soap.genomics.org.cn/soapdenovo.html) and the separated plasmid contigs of the 3 *mcr-1*–carrying plasmids from chromosomal contigs and compared them with pHNSHP45–2 by using blastn (http://blast.ncbi.nlm.nih.gov/Blast.cgi) and BRIG (*11*). We then analyzed replicon sequence types of these plasmids by using the Plasmid MLST Database (http://pubmlst.org/plasmid/) and performed analysis and annotation of the partial sequence of *mcr-1*–carrying plasmids by using the RAST Server (*12*), ISfinder (https://www-is.biotoul.fr), BLAST (http://blast.ncbi.nlm.nih.gov/Blast.cgi), and Gene Construction Kit 4.0 (Textco BioSoftware, Inc., West Lebanon, NH, USA).

For the 3 isolates, we found *mcr-1* on derivatives of IncHI2-like plasmids, which also harbored IncF (F4) and IncFIB (B53) replicons. The 3 plasmids were named pHNSH36 (from strain SH36), pHNZ319S (from strain Z4P319S), and pHNSH138 (from strain SH138). IncHI2 plasmids, such as pHNSHP45–2, have been frequently associated with global spread of *mcr-1* genes (*3**,**13**,**14*).

**Figure 1:**
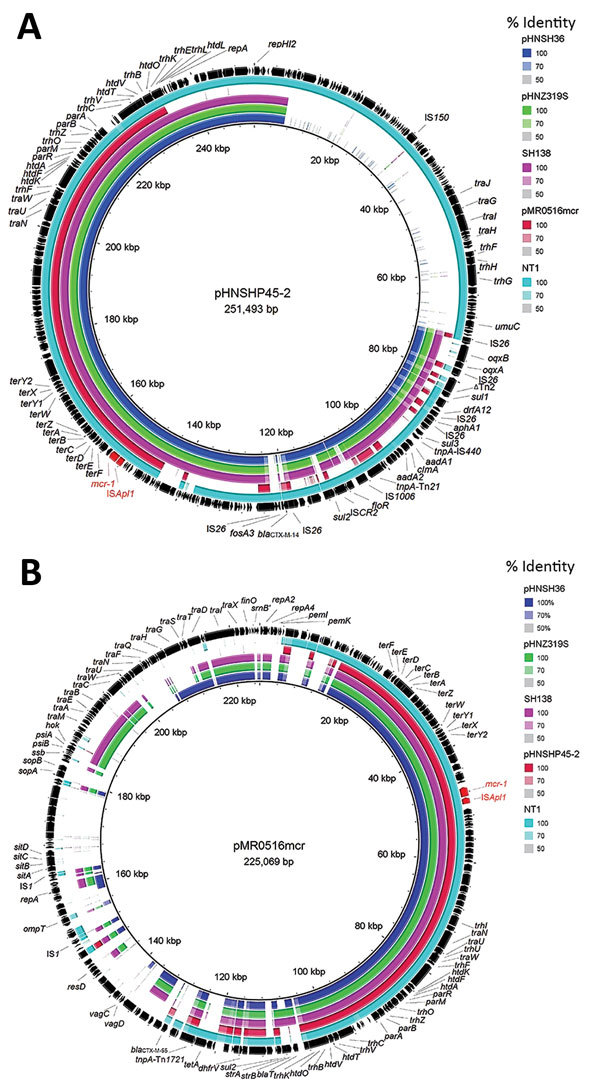
Sequence comparison of scaffolds (portions of genome sequences reconstructed from end-sequenced whole-genome clones) identified in *mcr-1*–positive plasmids pHNSH36, pHNZ319S, and pHNSH138 with 2 *mcr-1*–bearing plasmids pHNSHP45-2 (GenBank accession no. KU341381) and pMR0516mcr (GenBank accession no. KX276657), and contigs identified in *mcr-1*–positive genomes of *Escherichia coli* strain NT1 in BRIG (*11*) (GenBank accession LSBW01000090.1) obtained during analysis of *mcr-1*–positive *Salmonella* isolates from pigs at slaughter, China, 2013–2014. Arrows indicate positions and direction of transcription of genes. Reference plasmids pHNSHP45–2 (A) and pMR0516mcr (B) are indicated in black in the outer circles.

Compared with pHNSHP45–2 (in *E. coli* isolated from a pig in China [*14*]), the 3 plasmids all had a typical IncHI2-type backbone coding replication, transfer, maintenance, and stability functions, and a multiresistance region. However, we did not identify conjugative transfer region 1 of IncHI2 plasmids, including *traJGIH* and *trhRYXFHG*, in the 3 plasmids (Figure 1, panel A), which might be the reason why these plasmids were not transferred by conjugation.

**Figure 2:**
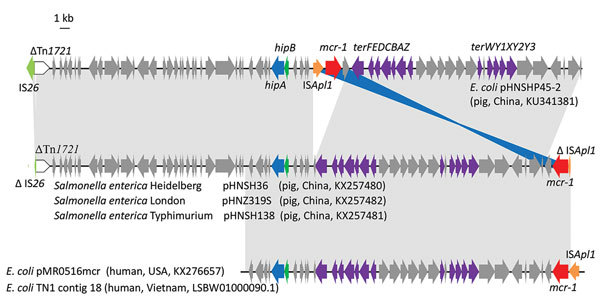
Genetic organization of scaffolds (portions of genome sequences reconstructed from end-sequenced whole-genome clones) containing *mcr-1* harbored by plasmids pHNSH36, pHNZ319S, and pHNSH138 obtained during analysis of *mcr-1*–positive *Salmonella* isolates from pigs at slaughter, China, 2013–2014, and structural comparison with plasmids pHNSHP45–2, pMR0516mcr, and *Escherichia coli* TN1 contig 18. Arrows indicate positions and direction of transcription of genes. Regions with >99% homology are indicated in gray or blue. Triangles indicate truncated genes. Information in parentheses after isolates indicate source, location, and GenBank accession number.

The multiresistance region contained numerous resistance genes, such as *oqxAB*, *floR*, *sul1*, *cmlA*, *aadA2*, and complete or truncated insertion sequences and transposons (IS*26*, ΔTn*2*, Tn*21*, IS*1006*, and IS*CR2*), which was similar to that of pHNSHP45-2, except that pHNSH36, pHNZ319S, and pHNSH138 were missing *fosA3* and *bla*_CTX-M-14_ (Figure 1, panel A). The 2,635-bp module (*mcr-1*-ΔIS*Apl1*) was similar to that of pHNSHP45-2, but different in some aspects, such as insertion sites and orientation, which were identical to those of the novel IncHI2-IncF plasmid pMR0516mcr (GenBank accession no. KX276657) found in a clinical *E. coli* isolate from the United States (*15*), and human *E. coli* strain TN1 from Vietnam (GenBank accession no. LSBW01000090.1) (Figure 2).

Similar to pMR0516mcr (IncHI2-F18:A-:B1) (Figure 1, panel B), the 3 plasmids also contained an IncF-type backbone, including regions coding replication and partial regions coding transfer, maintenance, and stability functions. Similar to pHNSHP45-2, the plasmid carrying *mcr-1* in strain NT1 belonged to the ST3-IncHI2 plasmid group. However, pMR0516mcr and the 3 plasmids described in this study were not typeable because there were no smr0199 loci in the IncHI2 plasmid (Figure 1, panel B). These data suggest that the *mcr-1*-IS*Apl1* module might have first been inserted into IncHI2 plasmids and that recombination between IncF- and IncHI2-type plasmids might have occurred subsequently, resulting in acquisition of the IncF-backbone fragment and deletion of the IncHI2 transfer region 1 or combination with the replication region in some instances.

## Conclusions

We found that spread of *mcr-1* in pigs at slaughter in China was associated with clonal dissemination of *Salmonella* Typhimurium ST34. This organism has also been detected in Portugal and England (*3**,**4*). The presence of indistinguishable IncHI2-F4:A-:B5 plasmids in different *Salmonella* serovars indicates that horizontal transfer of *mcr-1–*harboring plasmids might have also contributed to spread of *mcr-1* in *Salmonella* spp.

Other drug-resistance genes, such as *floR* and *oqxAB*, were always transferred with *mcr-1* by IncHI2-F4:A-:B5 plasmids, which led to successful flow of *mcr-1*–harboring *Salmonella* isolates under various selective pressures because florfenicol and olaquindox are widely used in farm animals in China. In addition, spread of similar IncHI2-like plasmids and *Salmonella* Typhimurium ST34 clones carrying *mcr-1* in different countries highlights the need for internationally coordinated intervention strategies to limit its further dissemination.
